# Optimization of Conditions for Microwave-Assisted Extraction of Polyphenols from Olive Pomace of Žutica Variety: Waste Valorization Approach

**DOI:** 10.3390/antiox12061175

**Published:** 2023-05-29

**Authors:** Ana Marđokić, Angela Estefanía Maldonado, Katalin Klosz, Máté András Molnár, Gyula Vatai, Szilvia Bánvölgyi

**Affiliations:** Department of Food Process Engineering, Hungarian University of Agriculture and Life Sciences, H-1118 Budapest, Hungary; maldonado.espinel.angela.estefania@stud.uni-mate.hu (A.E.M.);

**Keywords:** microwave-assisted extraction, olive pomace, total polyphenols, antioxidant activity, waste valorization

## Abstract

Olive pomace is a by-product of olive oil production that is toxic to the environment. The purpose of this study was to evaluate the methods of olive pomace valorization through the implementation of novel technology, the so-called microwave-assisted extraction process. To determine the total polyphenol content (TPC) and antioxidant activity (AA), polyphenol extraction using MAE was performed. Response surface methodology was used to determine the best extraction conditions, whereby the effects of three factors, solid ratio (g/50 mL), time (s), and power (W), were measured. The ferric reducing antioxidant power (FRAP) method was used to assess AA, whereas the spectrophotometric Folin–Ciocalteu (FC) method was used to determine TPC. The highest TPC of 15.30 mg of gallic acid equivalents per gram of dried weight (mg GAE/gdw) was generated after 105 s at 450 W, with a solid concentration of 1 g/50 mL, while the maximum AA was 10 mg of ascorbic acid equivalents per gram of dried weight (mg AAE/gdw). Numerical optimization revealed that 800 W, 180 s, and 1 g/50 mL were the best conditions for obtaining maximum TPC and AA.

## 1. Introduction

Due to growing concerns about environmental sustainability and its effects on nature, waste disposal from the olive oil industry has been the subject of much outstanding research in recent years [1,2]. It mainly affects the producing zone of the Mediterranean countries, from the coast of Portugal to Greece [3]. Various amounts of waste materials, such as solids and wastewater, are produced depending on the centrifugation technique used for oil extraction. In that regard, so-called two-phase centrifugation produces the largest amount of solid by-product, known as “alperujo”. This centrifugation technique is thought to be a more environmentally beneficial process because it uses less additional water when compared with three-phase centrifugation [4].

Alperujo is a semi-solid olive pomace residue with a moisture level between 55% and 65%, 9% to 19% carbohydrates, and 3% oil content [5]. Among the several options for the valorization of this waste, it is important to mention that it is a valuable source of oligosaccharides in the form of lignocellulosic materials, which makes it suitable for the extraction of dietary fibers [6] or pectic polymers, such as xylans, xyloglucans, and means [7]. Furthermore, the carbohydrate content makes alperujo a valuable source of fermentable sugars, which can be employed in cell fermentation to produce targeted products or anaerobic digestion to generate biogas [8,9]. It can also be employed for animal feed, contributing to completing a suitable nutritional profile [10], or as soil fertilizer because of its high content in potassium and nitrogen (19–24 g/kg and 10–18 g/kg, respectively) [11]. However, perhaps the most relevant feature of olive pomace is the number of polyphenol compounds [12], which can range from 2.9 to 3.7 g/kg. In olive pomace, hydroxytyrosol, tyrosol, and oleuropein are the three polyphenols that are most prevalent. However, some authors have also mentioned comselogoside, oleoside riboside, apigenin, and phenolic acids, including vanillic, homovanilic, and p-hydroxybenzoic acids [13,14]. The presence of these compounds represents an issue for valorization techniques, as the pH of the material is approximately 5. This makes alperujo a toxic waste material for soil irrigation and an inhibitor of microorganism growth in fermentation or anaerobic digestion [13]. Furthermore, it is necessary to remove these compounds to achieve viable concentrations of oligosaccharides [14].

Nonetheless, polyphenols are well known for their health benefits, such as their antibacterial, anti-inflammatory, and antioxidant properties [15]. Moreover, the solid antioxidative properties of polyphenols are known to help prevent various stress-related disorders, including cancer, cardiovascular diseases [16], kidney and liver diseases, and Alzheimer’s disease [17], as well as oxidative-related diseases of the human ocular surface epithelium [18]. Furthermore, the value of phenolic compounds as potential compounds for use in various industries [19], such as pharmaceutical, food, energetical, and chemical, has gained attention [20,21]. In that context, as bioactive compounds, polyphenols may become a “bottleneck” in functional food products, as they promote healthy constituents [22] and because eating foods rich in naturally occurring health-beneficial ingredients is becoming more widespread [23]. Additionally, there is proof that phenolic compounds from olive fruits and the by-products of processing can be utilized as natural antioxidants and antibacterial additions to enhance the preservation and nutritional qualities of food [24,25].

Recently, extraction techniques in the food industry have included new technologies. In a study by Chanioti et al. [26], different novel technologies, such as ultrasound-assisted extraction (UAE), homogenization (HAE), and microwave extraction (MAE), were studied to find the most suitable conditions for polyphenol extraction in a combination of different solvents. Compared to conventional procedures, MAE can accelerate the extraction process using less solvent [27,28]. The standard methods for extracting phenols from natural substances often involve organic solvents because they act as physical carriers for molecules moving between phases [29]. These techniques can be costly and time-consuming. Therefore, this study will investigate the impact of microwave-assisted polyphenol extraction from alperujo.

Numerous studies have already been conducted on microwave extraction of polyphenols from olive pomace using various solvents, including natural deep eutectic solvents [20,30], methanol [31], different olive cultivars [21], and experimental approaches [26], as well as conventional methods [32].

The Montenegrin olive variety Žutica is recognized for producing high-quality olive oil. It plays a significant role in the national olive industry and is receiving more attention regionally. To the best of our knowledge, this cultivar has no data available on the polyphenol content in its pomace. However, it is stated in the work of Adakalić et al. (2018) that the TPC in olive oil is 120 mg/kg [33].

In this work, microwave-assisted extraction was used to extract the polyphenol compounds in olive pomace from the Montenegrin variety Žutica. The main objective was to quantify and evaluate the polyphenol data obtained using the available data of other cultivars in the region and to determine the optimal conditions for extraction. Response surface analysis (RSM) was employed to establish the influence and best conditions of time (s), power (W), and solid ratio (g/50 mL) on total polyphenol content (TPC) and antioxidant activity (AA).

## 2. Materials and Methods

### 2.1. Chemicals and Reagents

For total polyphenol determination, Folin–Ciocalteu’s reagent (sodium 1, 2-naphthoquinone-4-sulfonate) and gallic acid supplied by Sigma-Aldrich (Budapest, Hungary) and sodium carbonate supplied by Merck KGeA (Darmstadt, Germany) were used. In addition, methanol at 80% supplied by Carlo Erba Reagents (Milan, Italy) and distilled water was used.

The reagents used for the ferric reducing ability of plasma method (FRAP method) were sodium acetate trihydrate, supplied by Lach-Ner (Neratovice, Czech Republic), hydrochloric acid (37%), supplied by Carlo Erba Reagents (Milan, Italy), iron (III)-chloride 6-hydrate puriss (ferric chloride), supplied by Reanal labor (Budapest, Hungary), and L-ascorbic acid, acetic acid, and 2, 4, 6-tris (2-pyridyl)-s-triazine (TPTZ), supplied by Sigma-Aldrich (Budapest, Hungary). All reagents were of analytical grade or higher.

### 2.2. Sample Preparation

Olive pomace (OP) was supplied by the olive oil factory ‘’Metović’’ (Bar, Montenegro). OP was obtained using the two-phase centrifugation technique. The sample was taken from the traditional Montenegrin olive cultivar Žutica, the most significant cultivar in the nation. For the experiments, olive pomace was brought to Budapest, Hungary, in a plastic container and kept in a freezer at −18 °C to prevent the degradation of polyphenols [21]. The sample was defrosted in warm water before being dried at 40 °C in a tunnel dryer until the moisture content was below 5% M. The initial moisture content was 59.22% M, while the percentage after drying dropped to 2.802% M. Moisture content was measured after each drying hour using a Kern MLS Moisture analyzer (Berlin, Germany). Final moisture was measured in triplicate. After the sample had been dried, it was additionally ground with a Knife Mill GM200 Grindomix Retsch (Berlin, Germany) to obtain a uniform structure and smaller particle size.

### 2.3. Microwave-Assisted Extraction of Phenolic Compounds

A prepared amount of dried and powdered samples (1 g, 3.5 g, and 6 g) was dissolved in 50 mL of ethanol solution at 52.7% and placed in a microwave oven (Specs Electrolux EMM 2005). Ethanol was chosen as the solvent because of its low toxicity and approval by the European Food Safety Authority (EFSA) for the creation of functional foods [26]. Its concentration was selected from the parallel experimentation of the research group as optimal for the performance of conventional extraction (not published results), while the described extraction methods were based on the experiment conducted by Zin and Bánvölgyi [34], with slight adjustments.

**Figure 1:**
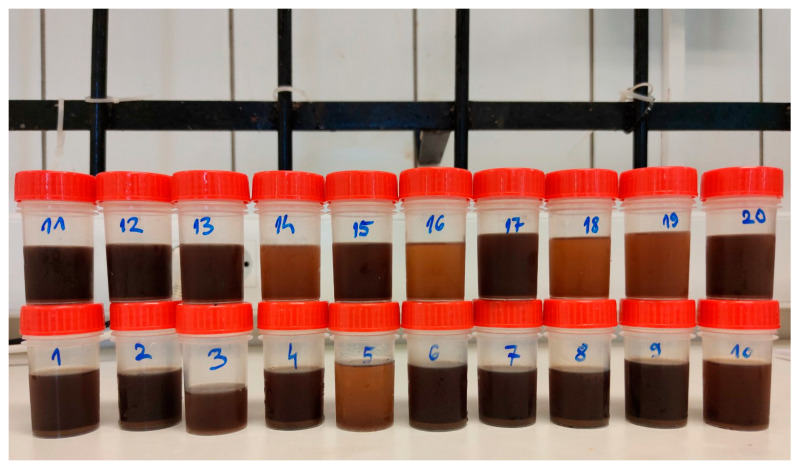
Olive pomace extracts obtained after microwave treatment.

For extraction at maximum microwave power (800 W), the intermittent mode was used for treating the pomace samples (10 s on, then 20 s off), with cooling between using iced water, which was found to be an effective method for the reduction of problems caused by direct microwave exposure [34]. This mode was optional for a shorter exposure period (30 s and 105 s) and with lower power modes (100 W and 450 W), since evaporation did not appear during testing. After microwave extraction, the extracts were filtered with a fine mesh sieve, poured into test tubes, and stored in a freezer until measurements (Figure 1). Before spectrophotometric analysis, the obtained extracts were centrifuged using a Z 206 A centrifuge for 10 min at 6000 rpm to remove the suspended solids from the sample.

#### Design of Experiment

The experiment was designed as a central composite design (CCD), requiring factors at three levels (−1, 0, 1). The effects of 3 varied factors, i.e., time (30–180 s), power (100–800 W), and solid ratio (1–6 g/50 mL), were investigated. The response variables were the total polyphenol content (TPC) and antioxidant activity (AA) of the extracts. Table 1 describes the independent variables and their levels.

Six center point replicates were included to estimate variability, with 20 extractions being the total. TPC and AA were analyzed in triplicate, which made 60 analyses in total. The randomized order of the tests that were run, along with the associated factor levels and their responses, are shown in Table 2. The variables were established based on a prior microwave extraction experiment carried out in a laboratory setting [34] with different sample materials and based on previous studies from a conventional extraction of polyphenols from olive pomace.

### 2.4. Determination of Total Polyphenols

The Folin–Ciocalteu (FC) colorimetric method is widely used to determine the total polyphenol content of plant-based samples [17]. Previously prepared olive pomace extracts were added to reagents according to the FC method, with slight modifications [34,35].

A calibration curve was prepared with 9 points, with gallic acid as a reference. In a test tube, 1250 µL of FC reagent was added, along with the concentrations of the methanol–water mixture and gallic acid. After exactly 1 min, sodium carbonate was added to obtain a total volume of 2500 µL and to stop the reaction. The mixture was shaken and placed into a thermal bath at 50 °C for 5 min. After this period, absorbance was measured at 760 nm against a blank probe with an HACH spectrophotometer DR2400. The obtained calibration curve had an R^2^ value of 0.97.

Similarly, absorbance at 760 nm was determined for each sample using the same steps as the standard calibration curve. A constant volume of 25 µL of the extracts was utilized for each measurement.

### 2.5. Determination of Antioxidant Activity

The ferric reducing antioxidant power (FRAP) method was used to determine antioxidant activity based on the method described by Benzie (1996), with slight modifications [36]. A buffer solution (solution 1) was prepared by mixing 3.1 g of sodium acetate trihydrate with 16 mL of acetic acid and then filling with distilled water up to 1 L. Similarly, 93.75 mg of TPTZ was added to 100.8 mL of HCl (37%) and filled up to 30 mL with distilled water, which yielded 30 mL of solution in total (solution 2). Ferric chloride solution (solution 3) was obtained by dissolving 162 mg of ferric chloride hexahydrate in 30 mL of distilled water. All three mentioned solutions formed FRAP reagents in 250 mL of solution 1, 25 mL of solution 2, and 25 mL of solution 3, which provides a total volume of 300 mL. The FRAP reagent was stored in a dark place and covered with foil until usage.

A calibration curve was prepared with ascorbic acid as the reference. Ascorbic acid (88.1 mg) was dissolved in 50 mL of distilled water and then diluted in a ratio of 1:10. In a test tube, 3000 µL of FRAP reagent was added to different volumes of ascorbic acid dilution and distilled water to complete 3100 µL. After exactly 5 min, absorbance was measured at 593 nm in different concentrations. The obtained calibration curve had an R^2^ value of 0.99. The same procedure was performed for the determination of antioxidant activity in the samples.

### 2.6. Statistical Evaluation

For a minimum of three independent experiments, data were given as the mean value, with a confidence interval of 2 times the standard deviation (*σ*), i.e., $\bar{x}$ ± 2*σ*. The standard deviation of TPC and AA were calculated with the model for error propagation shown in Equation (1), where *F* refers to Equations (2) and (3) for the expression of the results in grams of gallic or ascorbic acid equivalent per gram of dry weight (mg GAE/gdw) (mg AAE/gdw), and *x* refers to the variables of absorbance (*A*) and moisture (*M*):

(1) $$\sigma^{2}=\overset{n}{\underset{x_{i}=1}{\sum }}{\left(\frac{\partial F}{\partial x_{i}}\right)}^{\;2}\sigma_{x_{i}}{}^{2}$$

(2) $$\mathrm{TPC}\;\left(\frac{\mathrm{gGAE}}{\mathrm{gdw}}\right)=\frac{K_{1}A}{SR*m*\left(1-M\right)}$$

(3) $$\mathrm{AA}\;\left(\frac{\mathrm{gAA}}{\mathrm{gdw}}\right)=\frac{K_{2}A}{SR*m*\left(1-M\right)}$$

where *A* refers to the average value of the absorbance, m is the slope of the corresponding calibration curve, *SR* is the solid ratio of each experiment (g/50 mL), and *M* is the moisture of the pomace expressed as a mass fraction.

Mean values and correlation results were evaluated using Microsoft^®^ Excel. A demo version of Design-Expert^®^ Software, Version 13, performed the response surface analysis, optimization, and ANOVA analysis.

## 3. Results and Discussion

Table 2 contains the response values of TPC and AA for the 20 experiments. Values in the TPC example varied from 6.86 ± 0.33 to 15.30 ± 0.951 mg GAE/gdw, and values for AA ranged from 5.54 ± 0.70 to 10.00 ± 0.47 mg AAE/gdw. The results for TPC agree with the 7.08–18.30 mg/gdw reported by Chanioti and Tzia [26]. In that contribution, the phenolic compounds were measured using the HPLC technique after microwave extraction with deep eutectic solvents.

**Figure 2:**
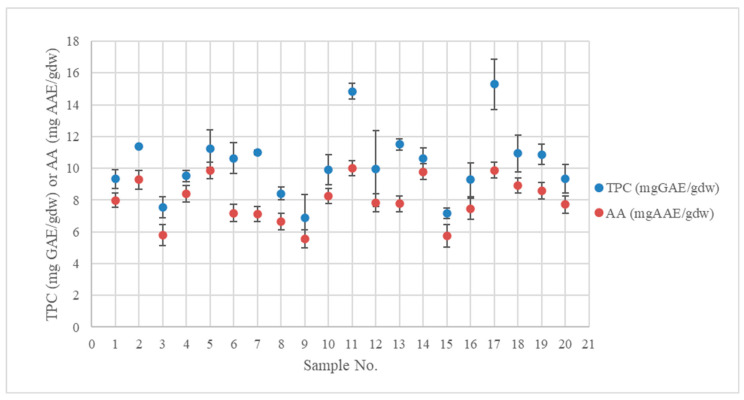
TPC and AA results obtained using the microwave extraction process.

There was a significant positive relationship between TPC and AA (r (18) = 0.812, *p* < 0.001), where r relates to Pearson’s coefficient. The findings thus demonstrate a considerable positive correlation between the antioxidant activity of the extracted polyphenols and their quantity. As a result, AA will increase proportionately to the amount of TPC in the sample (Figure 2).

### 3.1. Total Polyphenol Content

**Figure 3:**
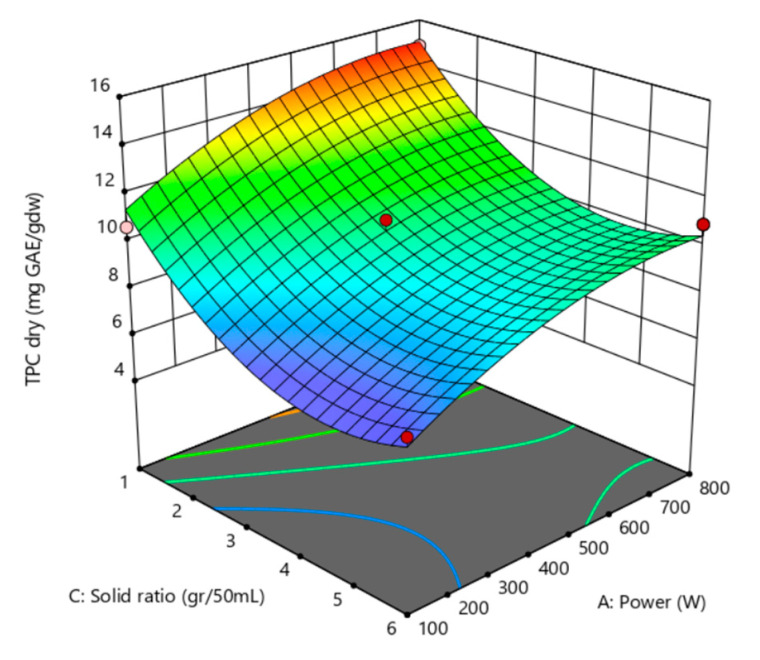
Surface response for TPC, presented with solid ratio (g/50 mL) and power (W), with time set at maximum (180 s).

Equation (4) represents the model’s equation, corresponding to a reduced quadratic model, with no significant interaction between variables. The correlation coefficient of this model was R^2^ = 0.886. A graphical representation of this model is presented in Figure 3, in which the characteristic curved shape can be seen at the end parts of the response surface graph showing the relationship between total polyphenol content (TPC) and the independent variables—power (W) and solid ratio (mg GA/gdw). This suggests that the TPC’s response to changing the independent variables was not continuous across the entire range of values.

(4) $$\begin{array}{c} \mathrm{TPC}\left(\frac{\mathrm{mg}\;\mathrm{GAE}}{\mathrm{gdw}}\right)=13.195+0.013\;*\;Power+0.000921\;*\;Time-3.571\\ {}*\;Solid\;ratio-8.466*10^{-6}*\;Power^{2}+0.379\\ \;*\;Solid\;ratio^{2} \end{array}$$

The model’s quadratic interaction between the power and solid ratio variables also points to an optimum within the evaluated range. The orange-to-yellow region in Figure 2 demonstrates the condition for the higher concentration of polyphenols—it suggests that increasing the power and solid ratio leads to an increase in TPC. This region has power intensities between 400 W and 800 W and the lowest solid ratios (1–1.5 g/50 mL). These requirements for optimal extraction are consistent with the argument made by Macedo et al. [37], i.e., a lower solid-to-liquid ratio increases the concentration gradient of the phenolic compounds between the pomace and the solvent, thus improving extraction efficiency. Additionally, higher power as a variable causes the solvent and pomace molecules to undergo more significant molecular excitation, which raises the diffusion rate.

Similarly, Tapia-Quirós changed the extraction time between 1 and 5 min concerning the influence of time and concluded that this aspect had no statistically significant effects [38]. However, higher TPC values of 9–50 mg GAE/gdw were reported by Jurmanović [15], with extraction times varying between 1 and 10 min.

The graph allows us to identify the optimal conditions for maximizing TPC. As presented in Table 2, the highest value of TPC in the experiments was obtained using 450 W, 105 s, and 1 g/50 mL of dried pomace, i.e., 15.29 mg GAE/gdw. However, numerical optimization of the model suggests that better results can be obtained at 800 W, 180 s, and 1 g/50 mL, where the maximum extraction is defined at 15.29 mg GAE/gdw.

The blue areas at the lower part of the graph show that TPC may decrease if the specific power and solid ratio criteria are exceeded.

### 3.2. Antioxidant Activity

To evaluate antioxidant activity, the model best adjusted to the data is presented in Equation (5), with R^2^ = 0.75.

(5) $$\begin{array}{c} \mathrm{AA}\left(\frac{\mathrm{mg}\;\mathrm{AAE}}{\mathrm{gdw}}\right)=6.56+0.004*Power+0.0039*Time-0.286\\ {}*Solids\;ratio \end{array}$$

**Figure 4:**
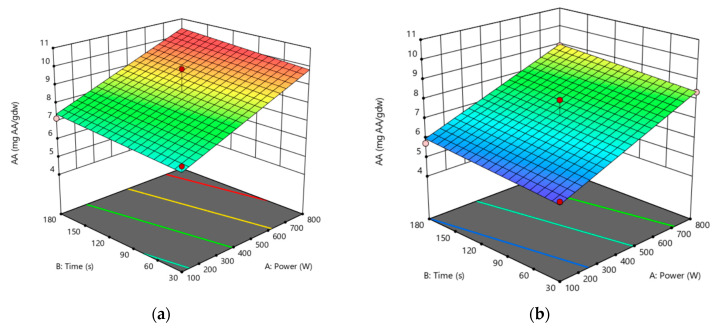
Surface response for AA, presented with time (s) and power (W), with (**a**) minimum solid ratio (1 g/50 mL) and (**b**) maximum solid ratio (6 g/50 mL).

Figure 3 represents the response surface plot for AA, with time (s) and power (W) as the descriptive independent variables. The antioxidant activity response surface provides a flat area, which is opposite in comparison to the curved-graph surface area with TPC. This suggests that changes in power (W) and time (s) within the range under consideration had little to no impact on AA. Therefore, the interactions between the components were insignificant in the linear model they were related to. Higher values of AA were obtained at a minimum solid ratio of 1 g/50 mL (Figure 4a), presented in orange, than at a maximum solid ratio of 6 g/50 mL (Figure 4b). Thus, the solid ratio factor significantly impacted antioxidant activity, which is consistent with the findings of Gómez-Cruz [39].

According to Table 2, the maximum value of 10.00 mg AAE/gdw was achieved at the high conditions of 800 W, 180 s, and 1 g/50 mL, corresponding to the numerical optimization of the model calculated at the exact upper limit of AA.

One of the main limitations when considering the solvent is the evaporation of ethanol during extraction, which occurs due to its polarity. A reaction medium’s temperature is quickly and uniformly raised by selecting the proper microwave settings, resulting in a higher reaction rate [40]. Different solvent blank probes in the function of time and power applied were carried out to approximate the evaporation trend. Testing revealed that the intermittent mode was only required when using 800 W continuously for an extended period (over 30 s).

As has been stated, another limitation could be the extraction time. It might be worthwhile to investigate longer extraction times, although the process’s economic balance would be challenging.

## 4. Conclusions

The olive pomace of the Montenegrin olive variety Žutica was utilized in this study of MAE to measure total polyphenols and their antioxidant activity. The described method showed a positive correlation between the concentration of polyphenols and their antioxidant activity. The maximum obtained result for TPC concentration was 15.30 ± 0.95 mg GAE/gdw, while the highest antioxidant activity was 10.00 ± 0.4710 mg AAE/gdw. The optimal conditions for maximizing TPC and AA were 180 W, 80 s, and 1 g/50 mL. The extraction efficacy was improved with a lower solid-to-liquid ratio. This practical strategy for using agricultural waste products can be scaled up to provide an additional source of income for olive farmers and olive oil manufacturers [39], but it can also serve as a database for polyphenol content in the olive variety Žutica, since the available data are scarce. Resulting from the findings, the Žutica variety has potentially high polyphenol content in olive pomace, but additional investigation is required to learn more. The study could contribute to research on the efficacy of MAE for green extraction by comparing the results with different techniques, not only conventional but also other new-technology methods, such as ultrasound-assisted extraction (UAE) [41], homogenization (HAE), and high hydrostatic pressure (HHPAE). However, more research and development are required to surpass their limits.

Additionally, information on the different compositions of the specific polyphenols detected in olive pomace for future research work could help to improve the current findings of this study. More detailed information could be provided by the quantification of polyphenols in olive pomace using HPLC-DAD with a reference compound. This approach can provide accurate measurements and enhance the understanding of polyphenol content. Hence, further research investigation could be oriented toward exploring and utilizing this quantification method to further advance the knowledge and application of specific polyphenols contained in olive pomace. Before performing the aforementioned analysis, a sample could be prepared using the extraction process indicated in this work.

To increase the stability of oils and other food products, it is important to use antioxidant extracts made from natural sources [42]. The results of this study contribute to our existing understanding of the polyphenols found in olive pomace that have antioxidant effects. The key characteristics of this work contribute to its scientific originality and possible improvements in polyphenol extraction.

## Figures and Tables

**Table 1:** Independent variables and their levels.

Variables	Low (−1)	Center (0)	High (1)
Power [W]	100	450	800
Time [s]	30	105	180
Solid ratio [g/50 mL]	1	3.5	6

**Table 2:** The resulting average values of TPC and AA and their factor levels.

No.	Power (W)	Time (s)	Solids (g/50 mL)	TPC (mg GAE/gdw)	AA (mg AAE/gdw)
1	450	105	6	9.32 ± 1.19	7.99 ± 0.53
2	800	105	3.5	11.35 ± 1.15	9.28 ± 0.49
3	100	105	3.5	7.55 ± 1.07	5.79 ± 0.67
4	800	30	6	9.52 ± 0.68	8.39 ± 0.51
5	450	30	3.5	11.24 ± 0.15	9.86 ± 0.47
6	100	180	1	10.64 ± 2.38	7.18 ± 0.57
7	100	30	1	11.01 ± 1.47	7.13 ± 0.57
8 *	450	105	3.5	8.42 ± 0.05	6.66 ± 0.66
9	100	30	6	6.86 ± 0.33	5.54 ± 0.70
10 *	450	105	3.5	9.92 ± 0.41	8.26 ± 0.52
11	800	180	1	14.85 ± 0.61	10.00 ± 0.47
12 *	450	105	3.5	9.97 ± 0.35	7.83 ± 0.54
13	800	30	1	11.51 ± 0.63	7.76 ± 0.54
14	450	180	3.5	10.60 ± 0.51	9.79 ± 0.47
15	100	180	6	7.18 ± 0.65	5.74 ± 0.68
16 *	450	105	3.5	9.27 ± 0.96	7.45 ± 0.55
17	450	105	1	15.30 ± 0.95	9.88 ± 0.47
18	800	180	6	10.94 ± 0.36	8.92 ± 0.50
19 *	450	105	3.5	10.87 ± 1.59	8.58 ± 0.51
20 *	450	105	3.5	9.35 ± 0.90	7.71 ± 0.54

* Center points of Central Composite Design (CCD). The data reported are the mean values of the triplicate measurements.

## Data Availability

Data is contained within the article.
